# An Automated Motion Detection and Reward System for Animal Training

**DOI:** 10.7759/cureus.397

**Published:** 2015-12-04

**Authors:** Brad Miller, Audrey N Lim, Arnold F Heidbreder, Kevin J Black

**Affiliations:** 1 Psychiatry, Washington University School of Medicine; 2 Electronics shop, Washington University School of Medicine; 3 Departments of Psychiatry, Neurology, Radiology, and Anatomy & Neurobiology, Washington University School of Medicine

**Keywords:** neuroimaging, neuroscience, positron emission tomography (pet), nonhuman primate, macaca fascicularis, video recording, operant conditioning, reward, computers, magnetic resonance imaging

## Abstract

A variety of approaches has been used to minimize head movement during functional brain imaging studies in awake laboratory animals. Many laboratories expend substantial effort and time training animals to remain essentially motionless during such studies. We could not locate an “off-the-shelf” automated training system that suited our needs.

We developed a time- and labor-saving automated system to train animals to hold still for extended periods of time. The system uses a personal computer and modest external hardware to provide stimulus cues, monitor movement using commercial video surveillance components, and dispense rewards. A custom computer program automatically increases the motionless duration required for rewards based on performance during the training session but allows changes during sessions. This system was used to train cynomolgus monkeys (*Macaca fascicularis*) for awake neuroimaging studies using positron emission tomography (PET) and functional magnetic resonance imaging (fMRI).

The automated system saved the trainer substantial time, presented stimuli and rewards in a highly consistent manner, and automatically documented training sessions. We have limited data to prove the training system's success, drawn from the automated records during training sessions, but we believe others may find it useful. The system can be adapted to a range of behavioral training/recording activities for research or commercial applications, and the software is freely available for non-commercial use.

## Introduction

Training animals for science experiments can be a time-consuming and labor-intensive process. Automated training systems reduce the labor commitment and have the potential to apply more consistent criteria and rewards than a human trainer who might be distracted, fatigued, or overwhelmed by demands of the training paradigm.

In many behavioral paradigms, an animal is required to complete a task in order to obtain a reward; for example, a bird might be required to peck a specific key. We faced the slightly different problem of training the animal to do nothing, or more accurately, to remain awake but motionless during the pharmacological challenge of fMRI (functional magnetic resonance imaging) and PET (positron emission tomography) brain imaging studies.

One purpose for imaging awake animals was because anesthesia might interfere with the function of the system of interest. Our study examined the role of a dopaminergic D_1_ receptor agonist on brain function, and anesthesia can alter regional cerebral blood flow responses to dopaminergic drugs in primates [1-2].

Two considerations motivated training the animal to remain motionless for our studies. First, even with a head restraint, body movement can produce slight head movements that can cause motion artifacts in brain images [3]. Second, all movements are initiated by brain activity, which produces changes in blood flow, metabolism, and blood oxygenation levels that lead to the signals detected by PET and fMRI [4-7]. A number of additional strategies have been used to reduce such artifacts, including adaptations of image acquisition or reconstruction, scanner hardware, or animal restraint systems [8]. 

This report describes an automated system using a charge coupled device (CCD) camera connected to a computer to detect motion and dispense rewards after specified durations of motionlessness. The experimenter set up the equipment and started the program, after which all other elements of the training sessions were controlled by computer software that provided stimulus cues, detected motion objectively, increased reward intervals as appropriate, administered rewards, and logged all of these events.

## Technical report

### Subjects

The subjects were five ~5-year-old male cynomolgus monkeys (Macaca fascicularis) housed at Washington University in a facility approved by the Association for the Assessment and Accreditation of Laboratory Animal Care. The monkeys were housed individually with 12-hour day/night cycles. All procedures used in this study met or exceeded National Institutes of Health guidelines and were approved in advance by the Washington University Animal Studies Committee (protocol #20050126). Licensed veterinarians monitored the animals’ health status. Measures were taken to minimize the number of animals used and to avoid animal suffering. Head motion during training was restricted either with a plastic cap attached to the skull or with a custom-molded thermoplastic mask.

### Plastic head cap

A plastic cap was surgically attached to the skull to limit head motion during brain scans [9-10]. Briefly, surgery was performed under deep anesthesia with 2.0% inhaled isoflurane and oxygen under sterile conditions. A polycarbonate post was attached to the top of the skull with nylon screws and dental cement. Six to eight weeks were allowed after surgery for the bone to heal around the nylon screws before the head post was attached to the chair during training.

### Thermoplastic mask

Mask Production:

After sedation with ketamine (10mg/kg i.m.) and intubation, anesthesia was maintained with 2.0% isoflurane and oxygen. Thermoplastic molding (Polyform®, Sammons Preston Rolyan, Bolingbrook, IL) was softened by placing it in a warm water bath. Strips of softened molding were applied to the animal’s head. A wide strip was shaped to the front of the monkey’s head, and a second wide strip was shaped to the back of the head. The ends of the strips were left as protruding tabs that were subsequently fastened with nylon screws to hold the two halves together securely around the animal’s head and to secure the mask to the training chair.

Mask Fitting:

After the mask was removed from the animal, eye holes were cut in the front of the molding so the animal could see the visual cues for training and testing. The mouth was left uncovered so the animal could receive liquid rewards. The mask was checked for fit and was easily reshaped by adding or removing material as well as by spot-reheating specific regions either with warm water or a blow dryer and physically reshaping the material. Foam padding was strategically added inside to enhance fit and comfort.

### Training paradigm

Animals were trained in a modified primate chair made of Plexiglas. Their heads were secured by attaching the head cap or thermoplastic mask to a bracket fixed to the chair. Animals were trained via operant conditioning to remain motionless in preparation for imaging sessions that would be up to 72 minutes long.

Light emitting diodes (LEDs) presented visual cues to the animal. A flashing green LED was the cue to hold still, and a solid red color LED provided feedback that movement was detected. When the animal moved, the computer resets the reward interval timer to zero. When the animal remained still for the specified duration, a liquid reward was dispensed. To prevent the movement associated with drinking from providing unwanted negative feedback during a 2-second “time out” period while the animal drank, the green light remained on but not flashing, the red LED did not light, and the timer was not reset.

Due to the shape and size of the MRI and PET scanner bores, animals were trained to accommodate a modified prone (“sphinx”) position [11]. Some researchers have reported success training animals without a gradual adaptation to the horizontal position, but at the time, colleagues had advised progressively changing the angle at which the primate chair was placed during a given training session from an upright (vertical) position to a horizontal position. Thus, animals were first trained in an upright position, and the incline was advanced by 30° increments after showing consistent adequate performance at a given angle.

Animals were introduced to the training system by being rewarded for remaining still for five-second intervals over several training sessions. After the animal earned 2-10 rewards at a given interval, the interval was increased. Initially, training sessions lasted about 30 minutes. Sessions were gradually extended to 60-80 minutes.

Desired behavior was rewarded with fruit juice, water, Kool-Aid®, peanuts, fruit, or candy. During the motionless training sessions, the automated system dispensed liquid rewards. Animals’ fluid intake was restricted to 20-30 ml/kg/day on workdays and withheld before training on the morning of each training session. The amount of fluid consumed during the training session was monitored and supplemental water was given after each session to ensure adequate hydration. On weekends, three-fold the weekday ration was provided. Body weight was recorded regularly and monitored by the veterinary staff to ensure adequate hydration.

### Training system: hardware

The computer was a Dell Optiplex GX280 with an Intel Pentium 4 CPU 3.20 GHz, 512 MB RAM, and an ATI Radeon X300 video card with 128 MB, running Microsoft Windows® XP Professional. We added a HuperLab H1004S video capture card (Huper Laboratories Co. Ltd., Taipei, Taiwan) and PC Witness Pro (DVR 2400) software (CCTV Wholesalers, New Orleans, LA). The card allowed smooth recording and display of video at 640x480 resolution at 30 frames per second (fps). A Sony SSC-M183 Super HAD CCD black and white video camera equipped with a Tamron 5-50mm 1:1.4 CCTV lens was connected to the capture card via a standard BNC cable.

[*Note: We subsequently adapted this system to a different experiment that required recording from two cameras. Recording two video feeds required a faster video capture card; the Huper Laboratories Co. Ltd. 2404Q-PCI interface card (purchased from Anova Microsystems, Inc., Milpitas, CA) allowed smooth video capture with two cameras at 640x480 resolution at 30 fps each.*]

Because the head was secured to the chair during training sessions, and to avoid detecting mouth/jaw movements associated with drinking the rewards, the video camera was directed toward the body, and if needed, extraneous portions of the camera's visual field were masked from motion detection using the PC Witness Pro DVR 2400 software.

**Figure 1:**
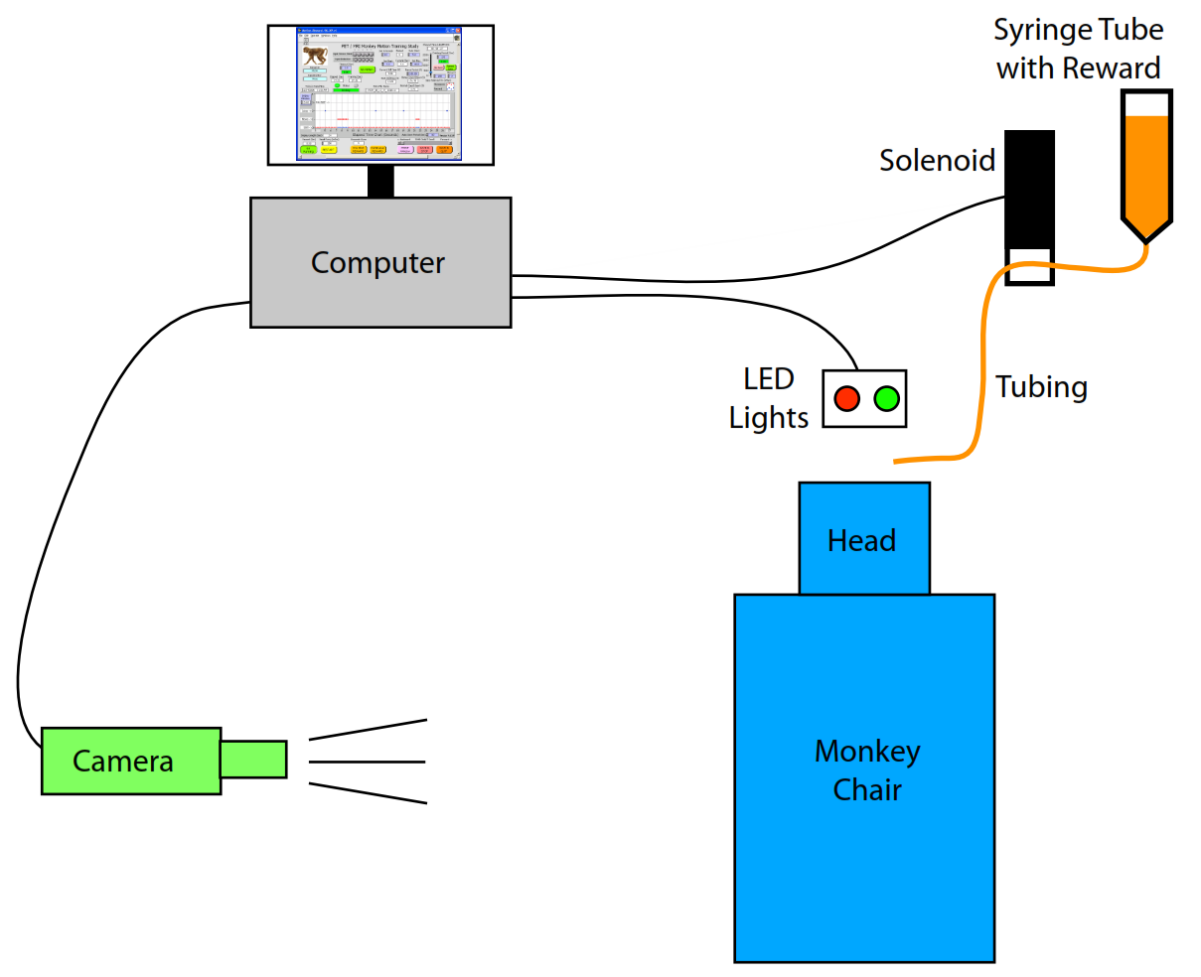
Illustration of the training system layout See text for details.

A 60 cc syringe (sans plunger) was used as a reservoir for juice rewards and mounted ~1 m above the animal’s head so that liquid was delivered by gravity. It was connected to flexible silicone rubber tubing that ran to the animal’s mouth through a solenoid-controlled pinch valve (Bio-Chem Valve Inc., part # 100P2NC12-05SQ, Boonton, New Jersey). The pinch valve closed the tubing by default and was activated (opened) to supply rewards. The duration of the reward flow was adjustable through the software’s control panel and a control box adjacent to the pinch valve. A hand-held push-button cable was connected to the solenoid valve control box to permit manual rewards. The physical arrangement of equipment in our training set-up is shown in Figure 1.

### Training system: Software

The automated training system involved a commercially available surveillance software program, PC Witness Pro DVR 2400 (then version 1.54), which detects motion in the video feed, integrated with custom software (Monkey Motion) written by one of the authors (AFH) using LabVIEW (National Instruments, Austin, TX, http://www.ni.com/trylabview/). The graphical source code file for Monkey Motion is freely available for non-commercial use at Zenodo.org (doi: 10.5281/zenodo.21157). Running this program requires the appropriate LabVIEW runtime software from National Instruments Corporation. Monkey Motion monitored an output signal from the surveillance software when motion was detected to control all other functions related to the training paradigm: the visual cues for the animal (LED lights), interval timing and reward administration. 

The stock DVR 2400 PC Witness Pro software (originally intended for security surveillance and recording) required a minimum event duration of 1 second to signal an event. At our request, the software programmers at Huper Laboratories under the direction of Geoff Wang modified the PC Witness Pro software to provide a nearly instantaneous electrical signal output to the custom LabVIEW software when motion was detected.

**Figure 2:**
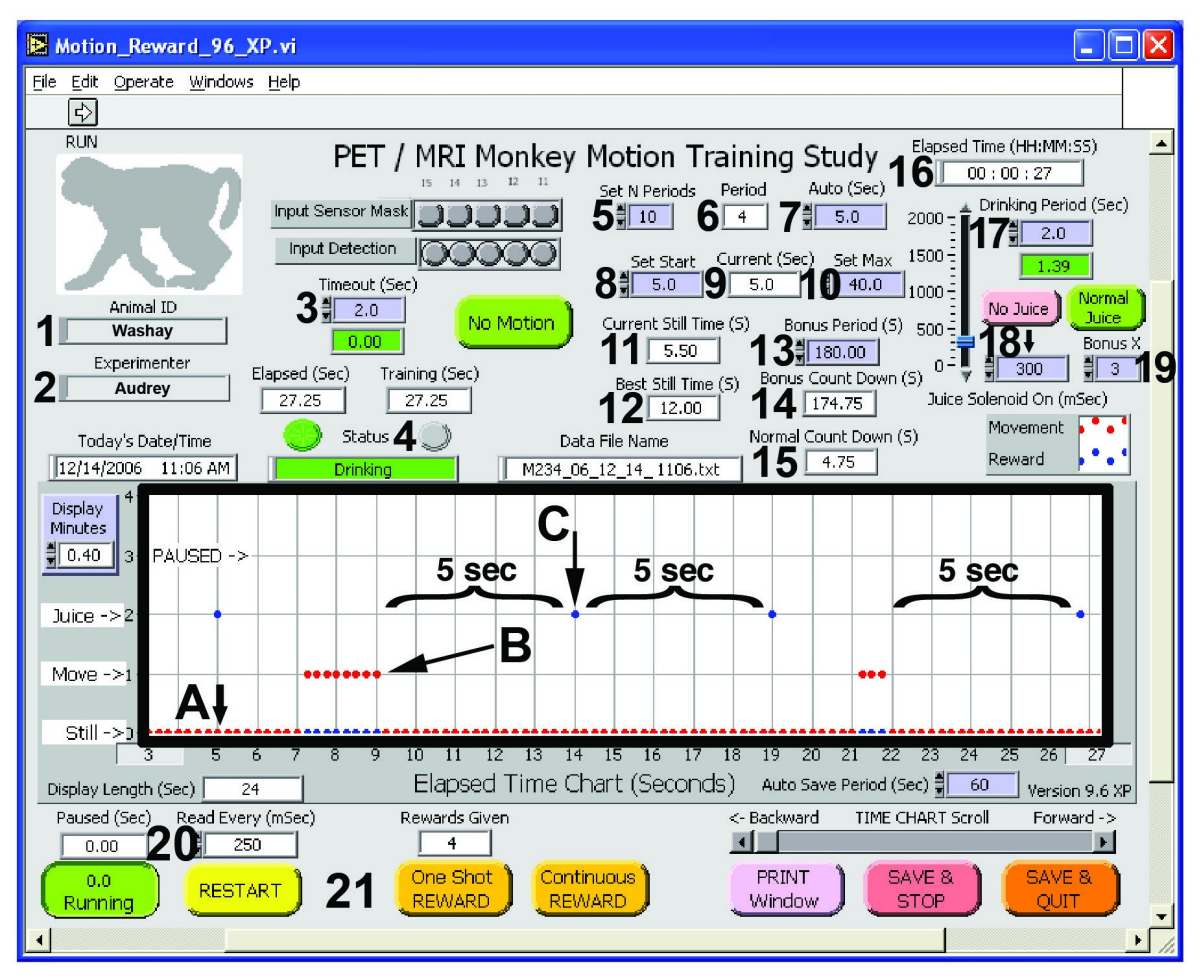
Screen shot of the Monkey Motion software interface with several features annotated by numbers, letters, braces, and arrows The black box shows a live record of the animal’s behavior (A = Still; B =  Moving), reward delivery (C), and the program’s status (running or paused). In this example, a reward is delivered automatically 5 seconds after the most recent movement (#9). Controls on a purple background can be set at program startup or during a session. Legend: 1. Animal ID. 2. Experimenter name. 3. Timeout. Time allowed for the subject to drink the reward, during which movement is not “detected.” 4. Status: No Motion, Motion, or Drinking. 5. Set N Periods: Number of consecutive earned rewards that triggers a change in the reward interval. 6. Period. Current number of consecutive rewarded periods. 7. Auto. Number of seconds the minimum reward interval will change by after the number of rewarded intervals set in #5. 8. Set Start. Initial reward interval (seconds). 9. Current. Current reward interval (seconds). 10. Set Max. Maximum reward interval period. 11. Current Still Time. A live count of the current time the subject has remained motionless (reset to zero when the subject moves). 12. Best Still Time. The longest period the subject has remained motionless in the current training session. 13. Bonus Period. Motionless interval required to receive an extra reward. 14. Bonus Countdown. Time remaining until the bonus reward. 15. Normal Count Down. Time remaining until the current motionless interval will be rewarded. 16. Elapsed Time. Duration of the training session. 17. Drinking Period. Time allowed for the subject to drink. 18. Reward Duration (milliseconds). Controls the size of the reward. 19. Bonus X. The size of the bonus reward as a multiple of the usual reward. 20. Read Every (mSec). Frequency of the sampling/recording intervals. 21.  Buttons on the bottom portion of the display allow the operator to pause, restart, give one reward or a continuous reward (for a duration longer than set by control #18), print the window, save and stop, or save and quit.

Figure 2 shows the front panel of the Monkey Motion program, with annotations explained in the figure legend. The active screen display shows when the subject is still, when a movement is detected, when a reward is given, and whether the program is paused. Additional displays on the window show convenient real-time information, such as current still time, best still time, and countdown time until the reward is given. Buttons on the front panel of the Monkey Motion program allow the user to start or pause training, give additional rewards, save data, or quit the program (Figure 2). The program records an ASCII text log file of all events and settings.

The LEDs were controlled via the parallel port. The parallel interface port was utilized as an integrated digital input and output signal port to receive and send TTL level signals for motion detected, visual indicators (LEDs), and the water/juice reward. The Monkey Motion software, written in LabVIEW, directly received and sent digital signals to this PC parallel I/O port. The H1004S indicated motion detection via the accessory I/O port located on the DVR card, which was directly connected to an input line on the parallel port. Specific output lines from the parallel port were then utilized to drive the red and green LEDs and the water/juice reward solenoid, appropriately interfaced with external DC power using power Field Effect Transistors (FETs). 

The numbers in boxes in the top portion of the software front panel (Figure 2) show elapsed time since movement and time to next reward, as well as the preset intervals for rewards. In general, controls shown in a purple background color can be set at program startup and modified, if desired, while the program is running. Timers are shown with a white background. The software allows setting the initial motionless period before a reward is given (Figure 2, #9). It also includes an adaptive feature whereby this period can be automatically increased or decreased after a given number of rewards (Figure 2, #5, #7). This automatic function assisted in training the animal to hold still for increasingly longer periods in preparation for the long PET and fMRI scan periods. It also allows a consistent training paradigm for each animal.

The valve-open duration (volume of the juice reward, Figure 2, #18) is also initially set by the experimenter; however, it can also be adjusted as the experiment progresses, and this is reflected in the data log file. An additional bonus reward criterion time is also set (Figure 2, #13) to give the animal an additional reward for longer cumulative periods of motionlessness.

**Figure 3:**
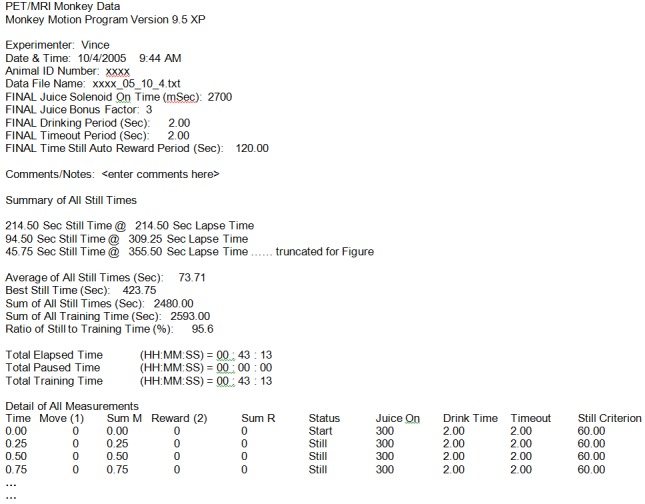
Example log file An excerpt from a text log file produced by the Monkey Motion program during one training session.

All training parameters, including Elapsed Time, Movement/Non-Movement, Status (START, STILL, REWARD, MANUAL, DRINKING, SENSED MOVE, TIMEOUT), Juice On (mSec), Drink Time (Sec), Time Out (Sec), and Criterion (Sec), are continually written at user-specified intervals to the data log file of that session (Figure 2, #20). The data log file integrity is protected during the long run of the experiment by periodically writing to a temporary data file on disk. At the end of the experiment, a summary of the performance throughout the training session is prepended to the log file and includes percent still time, the number of rewards that were given, duration of training time, longest “motionless” period, and starting and ending “motionless” interval criteria. Figure 3 shows an example of the output text file.

### Results

The data available to demonstrate the training system's success are drawn from the automated records created during training sessions. We show two sets of behavioral data.

### Results: Early training

**Figure 4:**
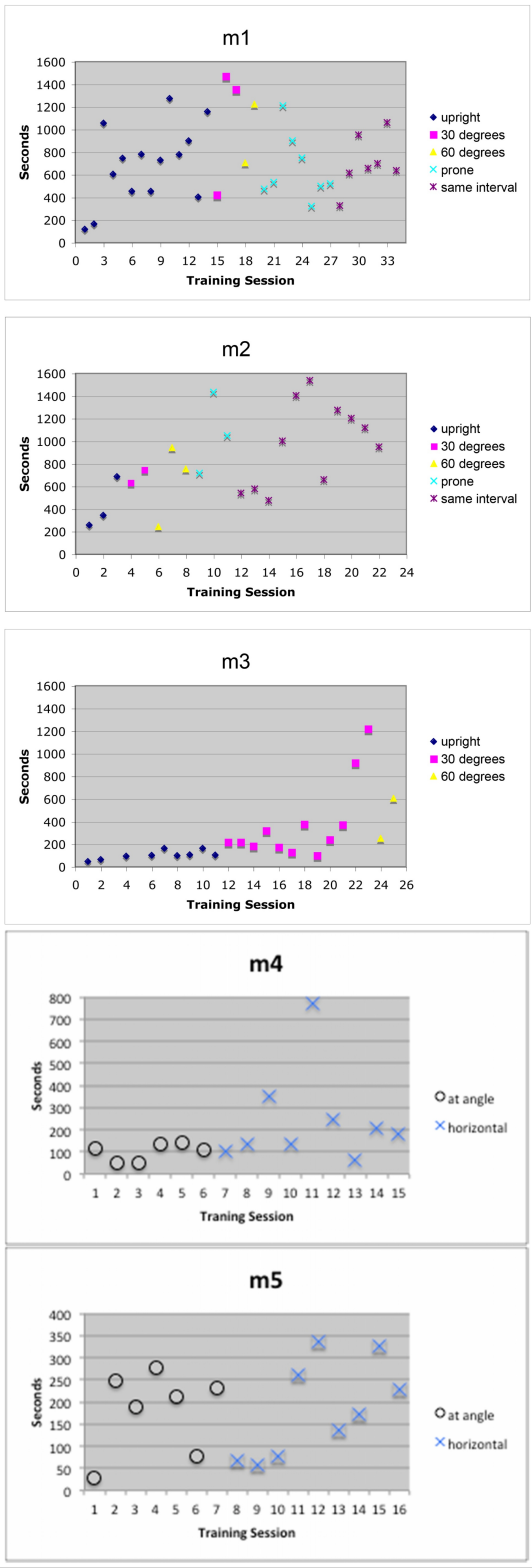
Early training results Different subjects' performance is plotted, in terms of the longest duration of motionless, during the transition from the training chair's being oriented vertically to being oriented horizontally. The marks labeled “same interval” on the graphs for m1 and m2 indicate sessions in which the period of motionless required for a reward was the same throughout that training session; the shortest interval used was 60 seconds, and the interval was gradually increased to 95 seconds for the last two sessions shown. For instance, the rightmost data point in the m1 graph comes from training session #34, during which rewards were given after every 95 seconds elapsed with no observed movement. This data point indicates that no motion was detected during a single period lasting over 10 minutes (discounting motion detected during 2-second breaks for reward delivery, so as not to discourage licking or swallowing motions.). The animal had additional still times during this session, but this 10-minute-plus interval was the longest.

The first data set shows the animals’ performance during the transition from training sessions in a vertical position to the horizontal position required for the brain scans. Previously, the monkeys had been habituated to working with humans, the primate chair, and the training room. The monkeys had also been trained with the LED visual cues. These data are from the early stages of training, while the automated computer system was being developed and before it was completely functional. During these sessions, the computer operated the LED visual cues and recorded movement and still intervals, but the human trainer was the motion detector and recorded the animal’s movement by pressing the space bar on the computer keyboard. The human trainer also gave the rewards manually. The “best motionless” period from each training session represents the longest single period that the monkey held still during a training session (ignoring movement while drinking), and is one of several measurements used to track an animal’s performance. The best motionless period from each training session during the transition from vertical to horizontal training is plotted in Figure 4. The *mean* duration of motionless periods over each animal's last six training sessions was 22, 27, 265, 23, and 19 seconds, one animal performing much better than the others.

Two animals (m1, m2) showed relatively rapid adaptation to the horizontal orientation. The performance of m1 began to decline once the chair was placed horizontally, but improved again after about 10 sessions. Performance declined briefly for m2 when the experimenter changed the starting interval (see description in Figure 4) but rebounded quickly. The third animal’s progress was slow. He showed gradual improvement over time and eventually improved with the chair at an angle, but his performance lagged far behind that of the other two monkeys, and his training was not completed. Two additional animals (m4, m5) showed relatively rapid adaptation to the horizontal orientation, but training data are available only for their initial training sessions, as by this time the grant funding the data collection was ending.

By the end of training, all animals were able to hold still the majority of the time. Over each animal's last six training sessions, the percent still time was 70%, 85%, N/A, 74%, and 84%.

### Results: Re-training

The second set of data shows the re-training of one monkey whose original surgically attached head cap loosened and had to be removed after several months of initial training. Training was interrupted for 14 weeks to allow the head cap site to heal. Subsequent training sessions and imaging sessions with this animal used a removable thermoplastic mask instead. The automated system was fully operational by this point and was used exclusively for subsequent retraining, automatically controlling all aspects of the training paradigm: visual cues, motion detection, interval recording, and reward administration.

**Figure 5:**
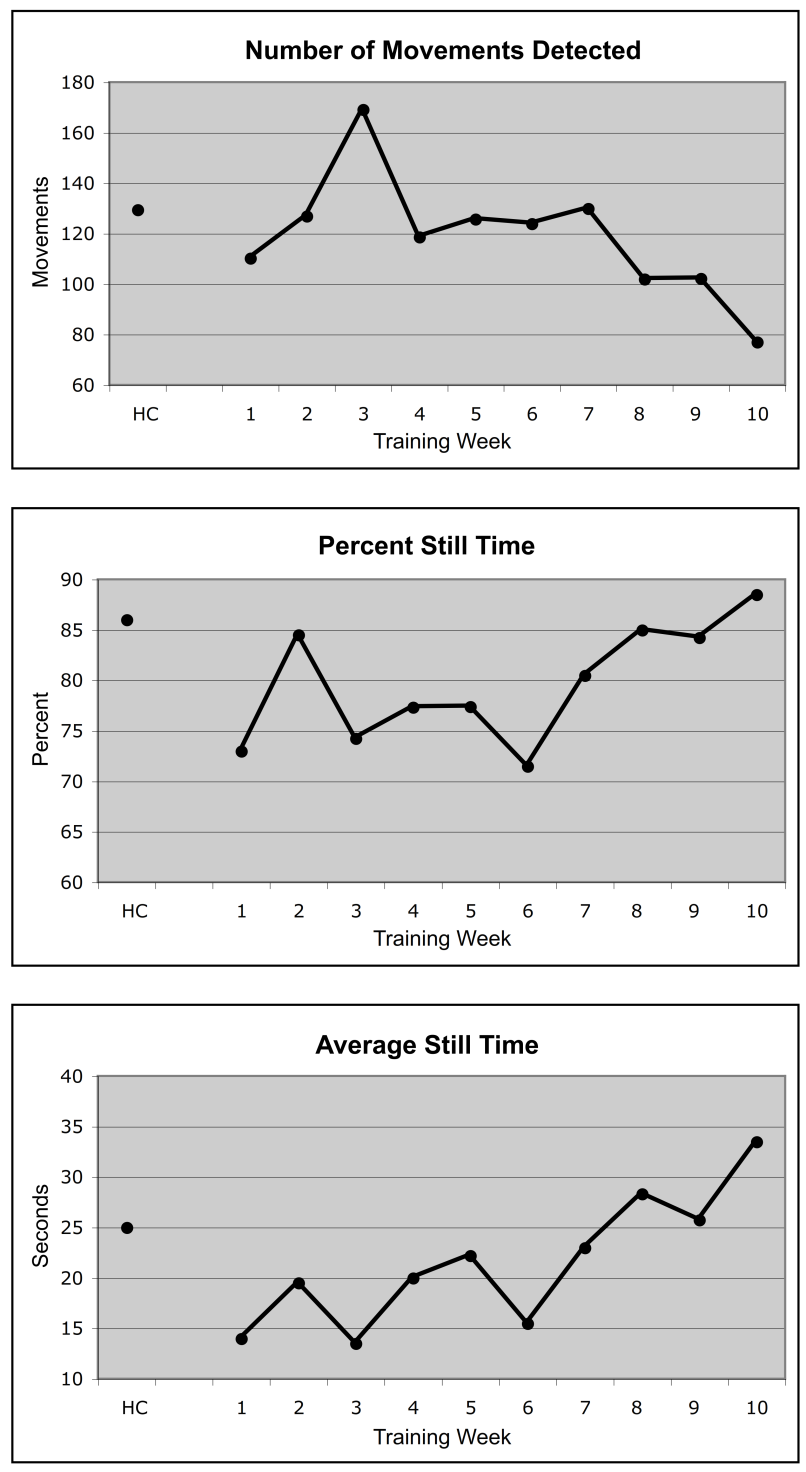
Re-training results This figure shows the performance of one animal that was retrained after a 14-week hiatus from training while his head cap was removed and he was subsequently fitted with a thermoplastic mask. The animal’s previous performance on these measures is shown by the single black circle above HC (head cap), representing the average of the data from the last two weeks of training prior to head cap removal. The connected points show the average performance for each week of retraining.

Figure 5 shows three different measures of this animal’s performance from the Monkey Motion program data logs: the number of movements detected, the percent still time, and the average cumulative still time. The animal’s performance on these measures while being retrained solely by the Monkey Motion system (connected dots in each graph in Figure 5) is compared with its performance before the head cap was removed (single dot at the left of each graph). The monkey’s performance on all measures at the end of 10 weeks of retraining equaled or exceeded its performance before head cap removal.

## Discussion

Prior to developing the automated training system, an observer was required to watch the animal for movements, mind and operate a stopwatch, and reward the animal when the animal held still for the designated duration. This training paradigm, although composed of relatively simple elements, required substantial effort from the trainer to apply consistent rewards in order to effectively shape the monkey’s behavior. Furthermore, judging training progress was fairly subjective. The programmable system virtually eliminated the need for human participation during the training procedures, except for the initial setup and to make sure that there was sufficient liquid in the reward dispenser.

The data presented show that the automated training paradigm effectively taught the monkeys to remain motionless for increasingly longer periods of time. The data also reflect individual differences in learning, even under highly reproducible conditions (Figure 4). The Monkey Motion system effectively maintained behaviors initially taught one-on-one by a human trainer (Figure 4) and quickly retrained one animal after a long absence from any training and with a new type of head restraint (Figure 5).

### Limitations

The data we present to document learning progress comes from the training sessions rather than from subsequent imaging sessions. Nevertheless, our imaging studies required the monkey to remain motionless for 40-minute fMRI scans and 72-minute ^18^F-fluorodeoxyglucose PET scans. The longest recorded motionless intervals were around 25 to 35 minutes. These durations are quite good for monkeys, considering that lying prone and motionless is not their typical behavior. Other researchers have reported that well-trained monkeys will sometimes fall asleep in their chair when not actively training [11], but that was not our experience at all.

Hardware costs for this system were about $2,300, and programming expertise was required to write the Monkey Motion program and tailor it to our specific needs. On the other hand, the automated system saved countless hours of hands-on training, and the session logs text file documented training progress continuously and objectively.

Our purpose in developing this system was primarily practical, so we did not directly compare the motion detection accuracy of the automated motion detection system with the accuracy of the human motion detector. We did, however, observe the animal during the sessions using the automated system and were satisfied at the time that it was detecting essentially the same movements we would have detected. Finally, our experience with this system did not include training older animals or females, or training without any physical head restraint, so performance in those situations may differ.

### Advantages of an automated system

The reduced animal/human contact has advantages for humans, such as fewer hazardous interactions with potentially dangerous animals. The automated training system appeared to provide advantages for the animal as well, including a more consistent presentation of visual cues, rewards, and less distraction by the activities of the human trainer. An additional benefit is the elimination of inadvertent experimenter bias between animals since the system ensures highly reproducible rewards. 

An additional advantage was observed when using the system during some brain scanning sessions to provide consistent rewards for holding still. Where the full setup could not easily be used (as in the MR suite), there was flexibility to use the Monkey Motion software without the camera with a human “motion detector” pressing a key to record observed movements. The record of animal movements and the timing of rewards was helpful for post-experiment data processing. 

This system has other potential uses, including training animals, recording behavior, monitoring patient activity, or any study in which movement is an experimental variable [12-14].

## Conclusions

We developed a time- and labor-saving automated system for animal training using readily available components to control and monitor various features of a behavioral paradigm that included providing stimulus cues, monitoring movement, and dispensing rewards. Although the system's components are now somewhat dated, and the proof of its efficacy is limited, we feel that this system, or portions of it, may be of use in a variety of behavioral training or recording activities for clinical, research, or commercial purposes.
